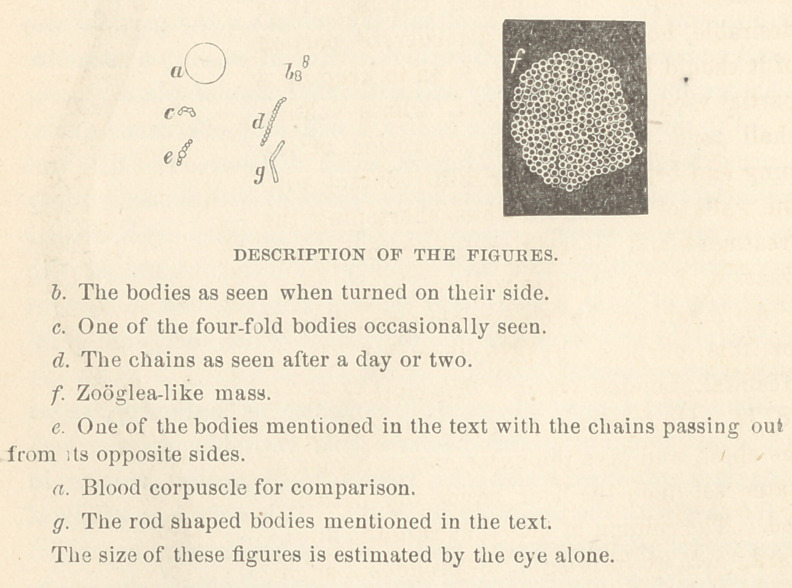# Micro Organisms Found in the Blood of a Case of Tetanus

**Published:** 1882-11

**Authors:** Lester Curtis

**Affiliations:** Professor of Histology in the Chicago Medical College; 1558 Wabash Avenue


### ARTICLE II.

MICRO-ORGANISMS FOUND IN THE BLOOD OF A CASE OF TETANUS. By Lester Curtis, A.B., M.D., Professor of Histology in the Chicago Medical College. Read before the State Microscopical Society, Oct. 27, 1882.

On Friday, July 22, I was asked by my friend, Dr. D. R. Brower, to see a case with him. The patient, a healthy German boy, about fourteen years of age, had received an injury with a toy pistol on the Fourth of July. About ten days afterward, symptoms of tetanus appeared. The disease had continued unabated up to the time when I saw him, and the prospects for his recovery were anything but favorable.

While in the house I drew a drop of blood from the patient's finger, taking precautions to avoid the entrance of foreign substances, and examined it immediately with one of Gundlach's high angled, homogeneous one-eighths, of recent construction. To my surprise, I saw a number of strange bodies in the preparation.

The bodies had a rapid dancing up and down motion. The motion was often unrestricted, but confined to a small space. Sometimes while dancing up and down they would have a circular swinging motion, like that of a top about to fall. They then appeared as though fastened by a short thread to something below.

Occasionally they would dart across the field, knocking against the blood corpuscles in their way until stopped by some obstacle, when they might be turned out of their course to dart across the field in another direction, or they might stop and begin again their dancing. They would sometimes be lost to view, either to be seen no more or to re-appear again after a time, and repeat over and over again their complex motions.

On account of their rapid motion, it was impossible to measure the bodies, but they were roughly estimated at about one-tenth the diameter of the red blood corpuscles. At first sight they appeared to be round, but after a time it was seen that many, if not all of them, were elongated and constricted in the middle. Their tendency was to stand on one end; only rarely were they seen lying on their sides, unless when they were moving across the slide. At this time, on account of their speed, it was difficult to determine their shape with certainty.

Several times I saw the bodies in the freshly drawn blood appearing four-fold instead of double. At this time they would be folded upon themselves and appear as irregular quadrilateral figures. Their specific gravity was lighter than that of the serum of the blood, so that their tendency was to float to the top of the preparation, while the heavier corpuscles sank to the bottom. Their number was not very great; usually not more than two or three were seen in one field of the microscope. It is probable that there were more of the bodies than were seen, for, as has been said, they frequently became lost to view among the corpuscles, and it was noticed that when the layer of blood was extremely thin and the corpuscles were separated by considerable intervals, the bodies were much more abundant than when the corpuscles were more crowded.

Besides these bodies the only specially noticeable peculiarities of the blood were a large number of very small red blood corpuscles, and a quite unusual number of fine pale granules. The granules were much smaller than the bodies that I have been describing, and often formed a complete layer between the corpuscles. They were pale, and seen with difficulty, and even then did not have a distinct sharp outline.

The observations were repeated on several different occasions, always with similar results. They were verified by Dr. Brower, and on one occasion by Dr. I. N. Danforth.

I carefully sealed up the preparations, so as to prevent the access of air and foreign matter, and took them home, where I could study them with a better light and better appliances than in the small bed-room of the patient. I then saw that the bodies had a tendency to grow. I also saw appearances as though they had cilia, though I cannot state positively that I actually saw such a structure. They seemed to have a tendency to attach themselves to objects in the field. Some of the blood corpuscles were surrounded by numbers of them. They were attached to the corpuscle by a pedicle. Occasionally I saw short chains formed of the bodies. These chains were sometimes floating free, sometimes one end was attached to a blood corpuscle, or some other object in the field. The chains were lashing actively. The joints in the chains seemed to be slightly smaller than in the dumb-bells first seen. In slides that had been kept for a day or so, the chains appeared to consist of ten or twelve links; but they never seemed to grow much longer.

I saw on one slide a large group of bodies somewhat larger than those first described. The last named bodies were uniform in size and circular in outline. They appeared to be flattened a little from above, downwards. They reminded me of zoöglœa masses of spores, but I am unable to say positively that they were anything more than masses of very small blood corpuscles. I have always, however, found these small corpuscles extremely variable in size, and I have never before seen so many in one mass.

On several occasions I saw a body which looked like one of these last, with a short chain of three or four links passing out from opposite sides. This appearance seems to me to have some connection with the growth of the chains, but I have not studied them long enough to be positive about the matter.

After keeping the slides for a few days, the long chains were not found, but there were seen large numbers of bodies joined in twos or threes, and still moving. I kept the slides for a number of days, and saw the bodies moving after more than a week had passed.

I will also mention that I found what appeared to be the same organisms in the blood of a horse suffering from tetanus, but as I failed to get the blood free from contamination with saliva and dust, the observation has little value except as suggestive.

I may, perhaps, be pardoned for being somewhat elated at this stage of my observations at the prospect of a new discovery. But I am suspicious of new discoveries, and in order to exclude the possibility of the bodies being due to local causes, I examined the blood of the mother and sister of the boy, who were living in the same house with him. The mother was nursing a small baby, and was, of course, much worn by anxiety and watching over a boy whom she supposed might die at almost any moment. Otherwise she was in good health, and her powers of endurance were unusual, for notwithstanding this strain, when she was relieved of her anxiety she soon became as fresh and well as ever. The sister appeared to be in perfect health. The blood of these persons, on examination, showed the same forms as were seen in the blood of the boy, and in about the same numbers. My air-castle was therefore dashed to the ground.

In time the boy recovered his usual health. Since his recovery I have examined his blood twice, at intervals of three or four weeks. When last examined the bodies were still present in about their former numbers.*

Looking around to discover, if possible, the source of these bodies, I noticed a pond of stagnant water in a vacant lot next the house. The pond contained organic matter, in a quantity and of the quality that duck-ponds in a city usually do. I collected some of this water, and found in it similar forms in great abundance, and in all stages of development.

The question that remains to be settled now is, what shall we consider these bodies to be? There are several sorts of bodies that might be found in this situation.

I cannot believe that they are foreign bodies. I have been for several years in the habit of studying blood from all sorts of people, and in all sorts of circumstances, often with no special precautions against the entrance of foreign matter, and have never before, except in perhaps one doubtful case, found any such bodies in the blood. Foreign bodies would scarcely have the uniformity of appearance and size that these bodies have, and we would expect them to be absent occasionally; or, at least to vary greatly in numbers. These bodies were remarkably uniform, both as to size and numbers.

Fat is also sometimes found in the blood, and it might be supposed that these were particles of fat. But particles of fat would not be likely to be so uniform in size, nor so constant in all the varying conditions of the patient, from that of severe and protracted illness to perfect health, and least of all would it be likely that the conditions for the production of fat in the blood should be so similar in persons so different in health as the three in whom the bodies were found. Fat also would have a refractive index not so near that of the blood serum, and it is inconceivable to me that fat should have the shapes that have been described.

It is sometimes possible to see in a white blood corpuscle, especially after it has been drawn from the body for some time, large numbers of granules. These are often seen to be moving. I have, in another place, said that "the movements of these gran-

* At the reading of this paper, the boy was present; blood taken from his finger at that time still showed the bodies, which were seen by most of those present.

ules seemed to be independent of each other, and reminded me of small animalcules imprisoned in a narrow space. By fixing my attention on one of them, and watching it for some time, I have seen it change its location and travel nearly half across the corpuscle before escaping from view." They may often be seen to escape from the corpuscle, sometimes singly, occasionally in great numbers; then they appear as granules in the blood. The general appearance of the granules resembles that of the bodies described, more than any other structure that has been mentioned, but differs from them in several respects.

The granules are single; although I have studied them for years, I have never seen them assume the dumb-bell shape. The refractive index of the granules is less than that of these bodies. And especially, however active their motion within the corpuscle may have been, they are always still, when seen free in the blood, as are all the bodies that have been mentioned. It is true that any or all of these bodies may have that peculiar motion called Brownian. This motion is often very evident if the particles are small and the layer of fluid is thick. But it is only a monotonous tremor without variety and without change of place.

As to the nature of these bodies, I will only offer some quotations. Nägle, quoted by Magnin, says: "There are but three distinctive signs which enable us to recognize with some certainty that granules under observation are organisms—spontaneous movement, multiplication and equality of dimensions, united with regularity of form. The most certain character is movement in a straight or curved line—a movement which inorganic granules never present."

Cohn, whom most of us will recognize as authority, says: "The distinction of pseudo-bacteria from veritable globular bacteria is a problem which our microscopists cannot resolve in every case with the desirable certainty. It is only by a study of their mode of development that this distinction can be made. The globules which divide and develop in the form of chains are organized beings. When this does not occur we are dealing with pseudo-bacteria."

Before leaving the subject, I will mention one other form that I have in a few instances seen in the blood after the slide had been kept for two or three days. This form consists of rods of nearly the length of the diameter of a blood corpuscle. The rods were usually joined in pairs, sometimes in threes. They were seen traversing the field, in the direction of their length, with a steady gliding motion, quite different from the jerky rapidity of the first bodies. They would often stop without apparent cause, and glide back again. Occasionally they were seen lying quietly at rest, when all at once, without any provocation, they would begin the motion that has been described.

Sometimes the moving ones would stop gently, either from coming in contact with some obstacle, or of their own accord, and remain at rest. As to the nature of these rods, whether they are a phase in the development of the bodies first seen, or whether merely foreign substances which effected an entrance while making the preparation I have not, up to the present time, been able to determine.

1558 Wabash Avenue.

## Figures and Tables

**Figure f1:**